# Design of a Magnetically Anchored Laparoscope Using Miniature Ultrasonic Motors

**DOI:** 10.3390/mi13060855

**Published:** 2022-05-30

**Authors:** Jingwu Li, Zhijun Sun, He Yan, Jinyan Chen

**Affiliations:** State Key Laboratory of Mechanics and Control of Mechanical Structures, Nanjing University of Aeronautics and Astronautics, Nanjing 210016, China; lijingwu3123@163.com (J.L.); 13218035117@163.com (H.Y.); jychen@nuaa.edu.cn (J.C.)

**Keywords:** laparoscope, ultrasonic motor, magnetic anchoring and guidance system, tilt motion, minimally invasive surgery

## Abstract

Images taken by an endoscope in single-port-access surgery are the most important information for directing surgeons to operate, so acquiring images taken at better position and a more desired perspective has profound significance for improving the efficiency and safety of surgery. The magnetically anchored laparoscope can help to realize this goal compared with the traditional laparoscope used in single-port-access surgery. In this paper, we propose the concept of applying ultrasonic motors in the magnetically anchored laparoscope. Two types of ultrasonic motors used for driving the laparoscope, namely a miniature traveling wave-rotating ultrasonic motor and a miniature traveling wave-tilt ultrasonic motor, are designed. The prototype of the magnetically anchored laparoscope using these two types of ultrasonic motors is fabricated and evaluated by experiments. The results show that the maximum output torque of the miniature traveling wave-rotating ultrasonic motor is 1.2 mN·m, and that of the miniature traveling wave-tilt ultrasonic motor is 1.4 mN·m, which is enough to actuate the magnetically anchored laparoscope. Additionally, it is proven that the two designed ultrasonic motors can be applied successfully in the laparoscope.

## 1. Introduction

Minimally invasive surgery that requires cutting only two or three holes in the human body has many advantages over traditional open surgery, such as less blood loss, less pain, shorter recovery time, lower risk of surgical recurrence, and better cosmetic results [1,2]. To further reduce the damage to the human body caused by minimally invasive surgical processes, a variety of single-hole surgical robot systems have been proposed [3,4]. This kind of surgical robot system only needs to cut one 25∼40 mm incision on the patient’s body [5,6]. Among these systems, the da Vinci SP Single-hole surgical robot system [7] has more mature technologies, and has been approved by the US Food and Drug Administration (FDA).

For single-hole surgical robot systems, all surgical devices are concentrated at the entry point, which will cause problems such as collisions and interferences between surgical instruments. At the same time, as the laparoscope is also located at the entry point, the position that the camera can reach and the shooting angle that can be achieved are restricted [8,9]. Images taken by laparoscopes are the most important information for directing surgeons to perform surgery, because surgeons operate surgical instruments based on their images. If the scope of motion of the laparoscopic camera is larger, then it will be possible to take images from more positions and at better angles to enhance the efficiency and safety of the surgery [10]. Thus, acquiring images taken at a better position and a great angle is significant.

The introduction of magnetic anchoring and guidance system (MAGS) enables the laparoscope to move freely in the abdominal cavity without having the abdominal wall fulcrum constrain [11], so that the laparoscope can move to a better position to photograph to acquire a better shooting effect. At the same time, the absence of the laparoscope at the opening of the abdominal cavity also alleviates the collisions between the surgical instruments and improves the performance of the single-hole surgical robot systems.

A number of researchers have carried out the study of developing an endoscope incorporating MAGS. Many developed MAGS endoscopes use electromagnetic motors to drive the motion of the endoscopes. Some researchers have introduced MAGS into the development of single-port surgical robot systems [12,13]. The laparoscopes in these systems have similar structures and the same driving mechanisms as other surgical instruments in the same systems. To improve the performance of laparoscopes, researchers have tried to give laparoscopes more functions, such as a laparoscope without line [14], in which the image data are transmitted to the console outside the abdominal cavity through Bluetooth; laparoscopes with lens-cleaning functions [15,16], which can remove blood stains and water vapor on the lens; and laparoscopes with a lighting optimization function [17,18].

The above-mentioned endoscopes are all equipped with electromagnetic motors to increase the degrees of freedom or improve the functions. Because the electromagnetic motor itself is large in volume, endoscopes equipped with electromagnetic motors are large in volume too. The electromagnetic motor also has the disadvantage of motion inertia, which results in a downgrade in control accuracy.

To avoid the drawbacks of using electromagnetic motors, researchers have designed a number of endoscopes that do not need to be integrated with motors. In this type of endoscope, magnetic force not only plays the role of moving and fixing the endoscope, but also controls the orientation of the endoscope. As early as 2007, the University of Texas [11] designed a magnetically driven endoscope, whose camera lens can be pointed upward and downward by changing the distance between the pair of external permanent magnets (EPMs) located outside the abdominal cavity. However, their later developed endoscope [19] can only reposition the endoscope by the EPMs, which lacks the ability to change the orientation of the camera lens. An endoscope with the ability to adjust its posture can take images from more angles, thus helping surgeons to perform surgery better. Therefore, researchers are also making efforts to develop endoscopes with higher dexterity. The endoscopes designed by Tan [20,21] or Cheng [22] are cylindrical, and the side walls of these endoscopes are in contact with the inner wall of the abdominal cavity. The endoscopes can tilt synchronously with the tilt motions of the EPMs. The endoscopes designed by Valdastri [23], Li [24] and Wei [25] can tilt arbitrarily within the hemispherical range by controlling the complicated external magnetic fields, and these endoscopes are more capable of moving. Another laparoscopic camera [26] with the local magnetic actuation mechanism utilizes the internal permanent magnets (IPMs) as the power source, and uses the cable-driven mechanism to transmit the power from the IPMs to the continuum structural manipulator. In this design, the internal permanent magnets play the role of motors.

MAGS endoscopes can be divided into two categories in terms of whether or not motors are installed. One is that the magnetic couplings between the EPM and IPM only have the functions of repositioning and/or fixing the endoscope; meanwhile, motors are introduced to provide power for change in posture of the endoscope. The degrees of freedom of these kind of endoscopes are determined by the number of motors used; the more motors installed, the more degrees of freedom the endoscope can have. The second category is for an endoscope that has no motor mounted on it. While using this type of endoscope, the magnetic force not only could anchor and translate the endoscope, but also has the function of changing the posture of the endoscope. Compared with the endoscope equipped with electromagnetic motors, the structure of this type of endoscope is simpler, and the volume is smaller. However, it has fewer degrees of freedom, a smaller range of motion, and less athletic ability.

When employing electromagnetic motors in the endoscope, its volume is large, but when not employing electromagnetic motors in the endoscope, its athletic ability is weak. To resolve this contradiction, we propose the concept of employing ultrasonic motors in the endoscope in this paper. An ultrasonic motor converts electrical energy into mechanical energy through the inverse piezoelectric effect of piezoelectric materials [27]. Compared with traditional electromagnetic motor, ultrasonic motor has the features of simple structure, stopping immediately after power failure. It is free from magnetic field interference, has low speed and high torque density, etc.

According to the different working modes of the piezoelectric materials, traveling wave ultrasonic motors can be classified into bonded-type and sandwiched-type motors. As early as 1988, SASAKI proposed a stator–rotor friction contact model of a traveling wave rotary ultrasonic motor. Maeno [28] put forward another stator–rotor friction contact model of a traveling wave rotary ultrasonic motor using the finite element method. The dynamic model developed by Mashimo calculates the transient response of the rotor accurately. The traveling wave ultrasonic motors have been applied in the field of robots, cameras, and other precision drive systems [29,30,31]. There is also a great deal of research about the applications of ultrasonic motors in the medical field [32,33,34,35,36,37,38,39].

The ultrasonic motor used for installation in the laparoscope should have the features of small size and high torque. To improve the torque density of an ultrasonic motor, Wu developed a rod-shaped transducer operating in torsional/bending modes, and the torque density of the ultrasonic motor was as high as 20 N·m/kg [40]. Feng proposed a ring-shaped traveling wave ultrasonic motor with a suspension stator; the stall torque of the ultrasonic motor was 49.5 mN·m [41]. A great deal of research about the sandwiched-type ultrasonic motor has also improved the output torque [42,43,44]. The output torques of these ultrasonic motors are high, but they are still too large to be used in the laparoscope.

In this paper, we designed two kinds of ultrasonic motors used for mounting in the magnetically anchored laparoscope. These two kinds of ultrasonic motors were applied to the magnetically anchored laparoscope, which can not only meet the torque requirements needed to actuate the laparoscopic joints, but also gives the laparoscope the advantages of smaller volume, no motion inertia, and no magnetic field interference.

The rest of this article is organized as follows. Section 2 details the design of two kinds of ultrasonic motors used for actuating the tilt and rotary motions of the magnetically anchored laparoscope. Section 3 presents the structure of the magnetically anchored laparoscope mounted with two kinds of ultrasonic motors stated above. Section 4 presents the control method of the laparoscope. Finally, the practicability of the laparoscope is verified by the experiments in Section 5.

## 2. Design of the Ultrasonic Motors

The two kinds of ultrasonic motors proposed in this paper are driven by way of superimposing two standing waves to form a traveling wave. Based on the design criterion [45] of the ultrasonic motor, we designed two types of ultrasonic motors: the micro traveling wave-rotating ultrasonic motor (MTWRUM) and the micro traveling wave-tilt ultrasonic motor (MTWRUM). The MTWRUMs are used to actuate the rotary and translating motions, and the MTWTUM is used to actuate the tilt motion of the laparoscope. The main consideration when designing the ultrasonic motors is to meet the torque requirements for driving the laparoscope, and at the same time, ensure the sizes of the ultrasonic motors are as small as possible, thus making the structure of the laparoscope more compact. The two kinds of ultrasonic motors are installed on the laparoscope, and the laparoscope can be inserted into the human body through a trocar with a diameter 15 mm.

### 2.1. Design of the Miniature Traveling Wave Rotating Ultrasonic Motor

**Figure 1:**
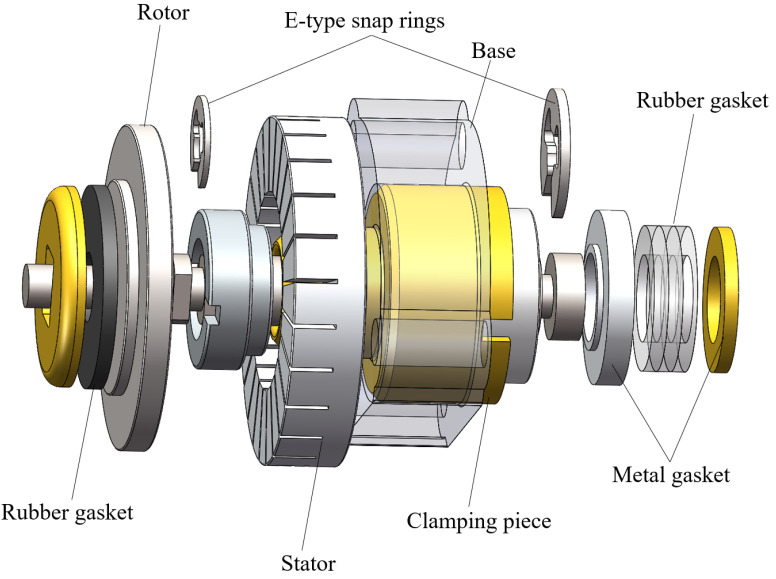
Structural scheme of the miniature traveling wave rotating ultrasonic motor.

**Figure 2:**
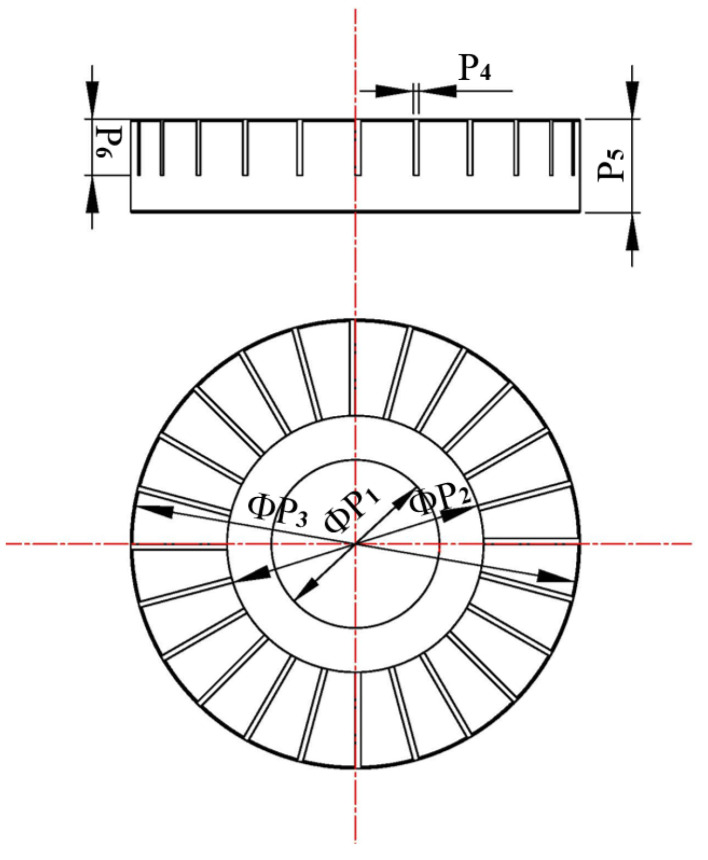
Structural dimension parameters of the stator.

The MTWRUM is mainly composed of a stator, a rotor, a base, and some accessories. Its structural scheme is shown in Figure 1. The stator, the base, and a clamping piece are assembled into a piezoelectric vibrator. The stator adopts a ring open-tooth structure, as shown in Figure 2, and the piezoelectric ceramic sheets are attached to the stator. Two E-type snap rings are mounted at both ends of the MTWRUM to achieve the axial positioning. There are several silicone rings with 0.5 mm thickness mounted before one E-type snap ring. The pretightening force between the stator and the rotor can be adjusted by changing the number of silicone rings. The pre-tightening force of the manufactured MTWRUM is 6.4 N.

**Figure 3:**
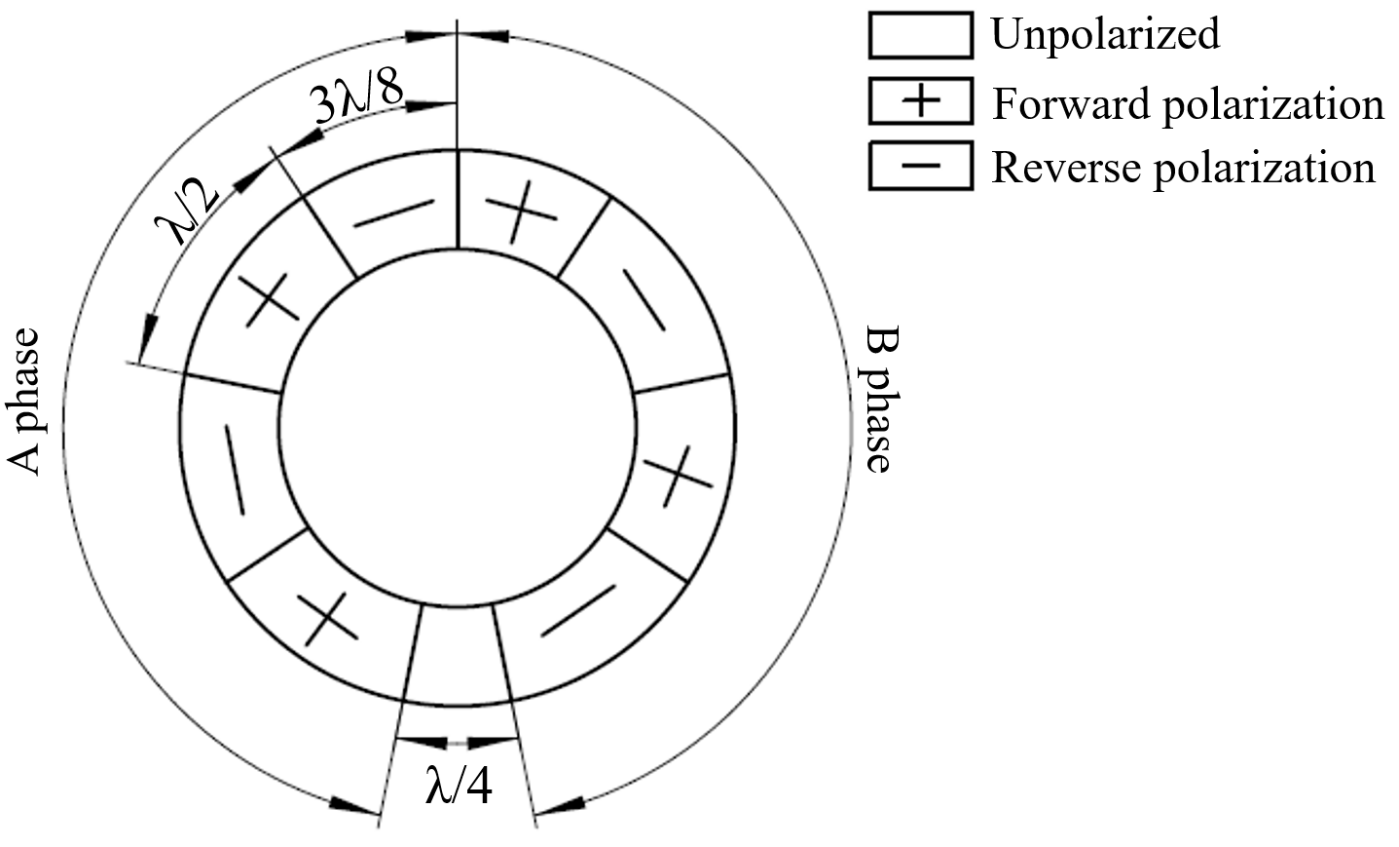
Stator polarization arrangement of the stator.

The stator is equipped with a circular piezoelectric ceramic element, which is polarized in the thickness direction, as shown in Figure 3. The unpolarized region of the circular piezoelectric ceramic element occupies 1/4 wavelength λ, which just satisfies the spatial phase difference π/2. The polarization regions at both sides form A and B phase, respectively. The manufacturing material is copper alloy. The piezoelectric ceramic sheets used in the MTWRUM are PZT-8 series with a thickness of 0.5 mm. The mechanical properties of aluminum alloy and piezoelectric ceramic sheets are shown in Table 1. Some other parameters of the piezoelectric ceramic sheets used for finite element analysis are listed below.

$$e=\left[\begin{array}{ccc} 0 & 0 & -5.2\\ 0 & 0 & -5.2\\ 0 & 0 & 15.1\\ 0 & 0 & 0\\ 0 & 12.7 & 0\\ 12.7 & 0 & 0 \end{array}\right]c/m^{2}$$

$$\varepsilon =\left[\begin{array}{ccc} 7.124 & & \\ & 7.124 & \\ & & 5.841 \end{array}\right]\times 10^{-9}F/m$$

$$C=\left[\begin{array}{cccccc} 12.06 & 5.35 & 5.15 & 0 & 0 & 0\\ & 12.06 & 5.15 & 0 & 0 & 0\\ & & 10.45 & 0 & 0 & 0\\ & & & 3.13 & 0 & 0\\ & & & & 3.13 & 0\\ & & & & & 3.46 \end{array}\right]\times 10^{10}N/m^{2}$$

where *e* donates the piezoelectric constant matrix, ε donates the dielectric constant matrix, and *C* donates the elastic stiffness constant matrix.

The vibration modal of the stator is out-of-plane bending mode, which is expressed by $B_{mn}$, where m and n indicate nodal circles and nodal diameters, respectively. To meet the requirements that the working modals of A and B phases should be as similar as possible, and the vibration amplitudes of both A and B phases need to be greater than 1 μm, we conducted the vibration modal analysis of the stator using Ansys, and continuously optimized the stator dimension parameters according to the simulation analysis results. If the inner diameter of the stator is too small, the excited energy will be consumed on the inner support of the stator, and if the inner diameter is too large, the output power of the MTWRUM will be small. The sizes of teeth of the stator also have a great influence on the output performance of the MTWRUM. When increasing the tooth height, the rotary speed will increase. However, if the tooth height is too high, the output torque will decrease. The final stator dimension parameters are shown in Table 2. The vibration modal is $B_{04}$ modal.

**Figure 4:**
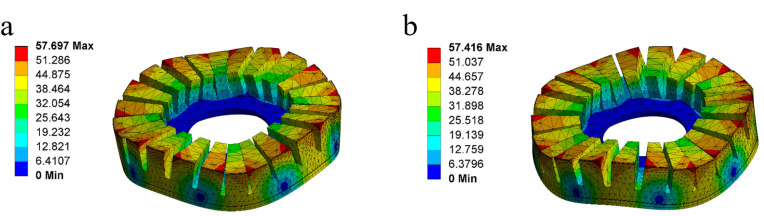
Vibration modals of the stator. (**a**) A phase; (**b**) B phase.

**Figure 5:**
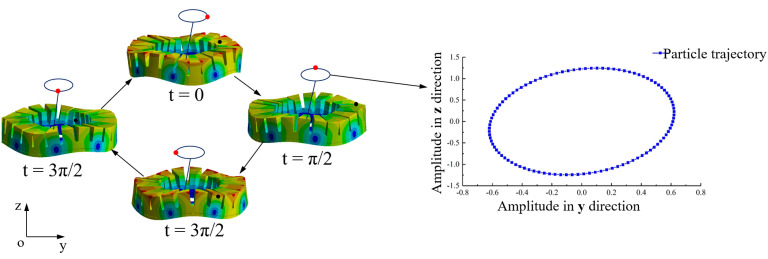
Vibration modal diagrams of the stator in one period.

Applying two sinusoidal excitation signals with a phase difference of π/2 to A and B phases of the stator, these two phases are excited into $B_{04}$ out-of-plane bending modals. The vibrations produced are standing waves. After superposition, two standing waves of A and B phases will form a bending traveling wave on the stator surface, which will drive the rotor to rotate by friction. The working modal of the stator is simulated, using the parameters shown in Table 2, as shown in Figure 4. The vibration frequency of A phase is 66.229 kHz, and that of B phase is 66.298 kHz. The frequency difference between A and B phases is 59 Hz, which meets the criteria that the vibration frequencies of both phases are consistent. Applying sinusoidal and cosine voltage excitation signals to A and B phases, respectively, the motion of a point on the stator in one cycle is shown in Figure 5. The vibration amplitude is greater than 1 μm, and it will move along an elliptical path.

### 2.2. Design of the Miniature Traveling Wave Tilt Ultrasonic Motor

The degree of freedom of the tilt motion is of great importance to a laparoscope. If using MTWRUM to drive the tilt motion of the laparoscope, the size of the laparoscope will increase, and the laparoscope cannot be inserted into the abdominal cavity through a trocar with a diameter of 15 mm. At the same time, a relatively complicated structure needs to be designed to complete the change in rotary direction. In this paper, a special type ultrasonic motor, a miniature traveling wave-tilt ultrasonic motor, is designed to drive the tilt motion of the laparoscope.

**Figure 6:**
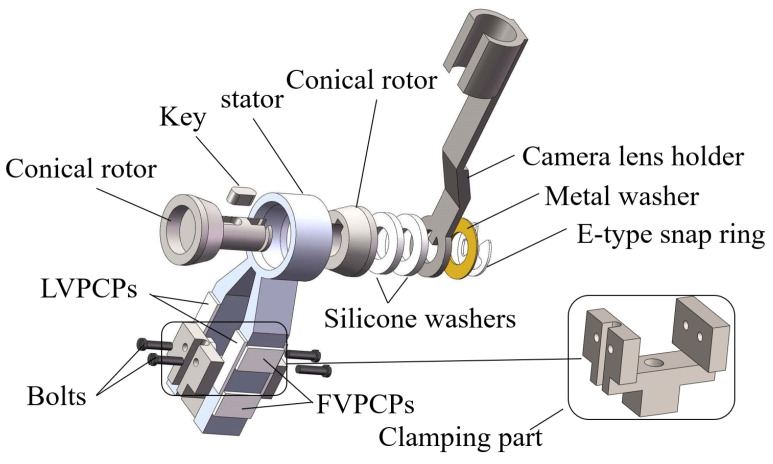
Structural scheme of the miniature traveling wave tilt ultrasonic motor.

The structure of the MTWTUM is illustrated in Figure 6. The motor consists of a stator, a rotor, a camera-lens holder, and some accessories. The MTWTUM adjusts the pre-tightening force between the stator and the rotor in the same way as the MTWRUM. The pre-tightening force of the manufactured MTWTUM is 14.5 N. The inner surface of the stator and the outer surface of the rotor are conical, which can increase the contact area between the stator and the rotor, thereby increasing the output torque of the ultrasonic motor. A camera is installed on the camera-lens holder that is fixed to the output shaft of the MTWTUM.

**Figure 7:**
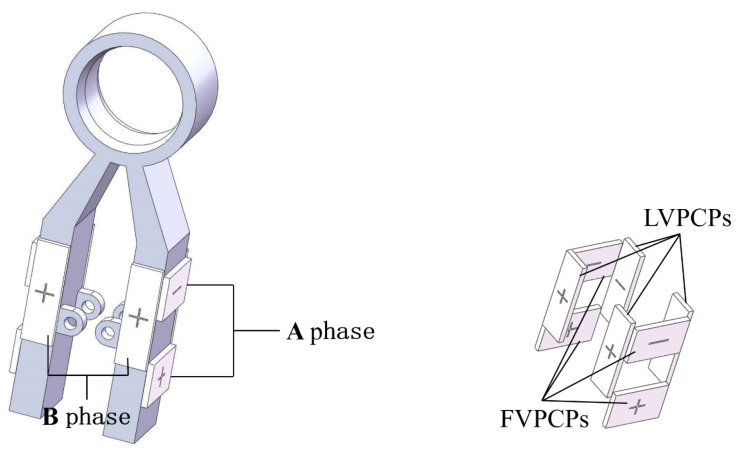
Structural scheme of the stator.

As shown in Figure 7, the stator has two legs. Each leg is attached with four piezoelectric ceramic pieces. Piezoelectric ceramic sheets are bonded to the stator by epoxy resin adhesive. All eight piezoelectric ceramic pieces are divided into longitudinal vibration piezoelectric ceramic pieces (LVPCPs) and flexural vibration piezoelectric ceramic pieces (FVPCPs) with four pieces in each group, polarized along the thickness direction, and forming A and B phases, respectively. LVPCPs are located at the sides of two legs. Their expansion and contraction will excite the longitudinal vibration of the stator legs using the Poisson effect. FVPCPs located at another two sides of each leg will excite the flexural vibration.

**Figure 8:**
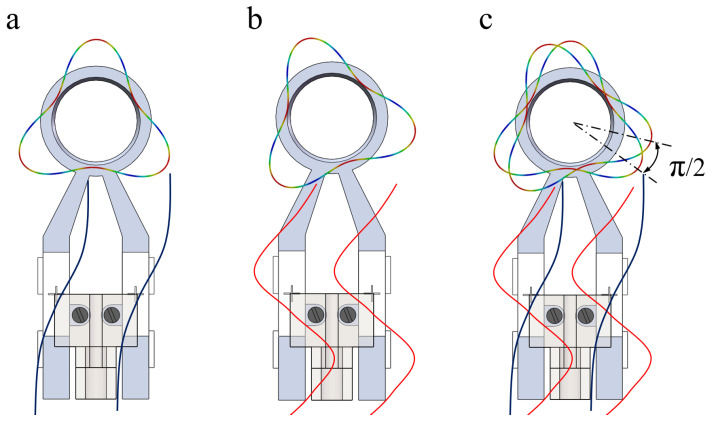
Vibration modals of the stator. (**a**) Is vibration modal of A phase; (**b**) is vibration modal of B phase; (**c**) is the combination of these two vibration modals.

When the excitation is applied to phase A of the stator, the first-order longitudinal vibration is excited in the stator legs, and the third-order bending vibration is excited in the ring of the stator, as illustrated in Figure 8a. Applying excitation to B phase of the stator, the second-order bending vibration is excited in the stator legs, and third-order bending vibration is excited in the ring of the stator, as illustrated in Figure 8b. The vibration function in polar coordinates is

(1) $$\left\{\begin{array}{c} \xi_{A}\left(\theta ,t\right)=W_{A}\sin\left(n\theta \right)\cos\left(\omega t\right)\\ \xi_{B}\left(\theta ,t\right)=W_{B}\sin\left(n\theta +\pi /2\right)\cos\left(\omega t+\varphi \right) \end{array}\right.$$

where θ is the angle variable, *t* is the time variable, $W_{A}$ and $W_{B}$ denote the amplitudes of phase A and phase B, respectively, ω denotes the angular velocity of vibration, and φ denotes the phase difference between the external excitations applied to phase A and phase B. When there are excitations applied to both A and B phases of the stator, the displacement response function superimposed by the two phases is

(2) $$\xi \left(\theta ,t\right)=\xi_{A}+\xi_{B}=\frac{1}{2}\left(W_{A}+W_{B}\sin\varphi \right)\sin\left(n\theta -\omega t\right)+\frac{1}{2}\left(W_{A}-W_{B}\sin\varphi \right)\sin\left(n\theta +\omega t\right)+W_{B}\cos\varphi \cos n\theta \cos\omega t$$

The simultaneous application of excitation signals to the longitudinal ceramic plates and the bending ceramic plates excites the first-order longitudinal vibration and second-order bending vibration in the stator legs, and then two third-order in-plane bending vibration modals are excited. When the timing phase difference of these two in-plane bending vibration modals is π/2, as shown in Figure 8c, a traveling wave rotating in the circumferential direction will be superimposed onto the inner surface of the ring of the stator. Then, the particles on the inner wall of the ring will move along the elliptical path. The Formula (3) can be simplified as

(3) $$\xi \left(\theta ,t\right)=W_{0}\sin\left(n\theta \pm \omega t\right)$$

**Figure 9:**
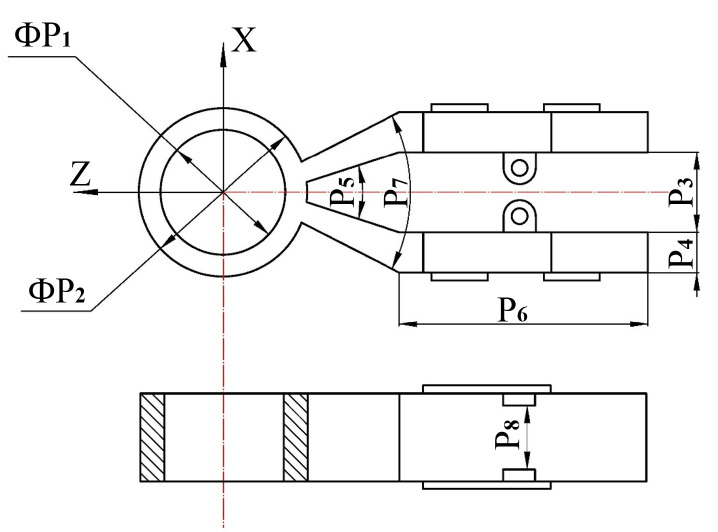
Structural dimension parameters of the stator.

The main structural dimension parameters of the stator are shown in the Figure 9. The vibration modal of the MTWTUM is simulated by Ansys, and the dimension parameters of the MTWTUM are constantly optimized, until phase A and B can generate two third-order bending modals with same frequencies and vibration amplitudes. The final structural dimension parameters are determined as shown in Table 3. Using these dimension parameters, the working modals of A and B phases are simulated. The results present that the vibration frequency of the first-order longitudinal modal is 125.23 kHz, and that of the third-order bending modal is 125.36 kHz.

## 3. Design of the Laparoscope

**Figure 10:**
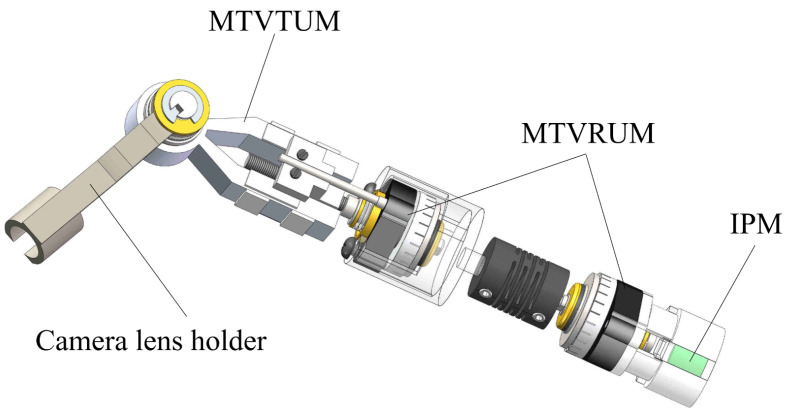
Structure of the laparoscope.

In order to verify the usability of the designed ultrasonic motors, we designed and manufactured a laparoscope equipped with the proposed ultrasonic motors, whose structure is shown in Figure 10. The IPM is mounted at the top of the laparoscope. The laparoscope will be fixed on the inner side of the abdominal wall through magnetic coupling between the EPM and the IPM. The ultrasonic motors mounted in the laparoscope can drive the motions of the laparoscopic joints to adjust the posture of the laparoscope, so that the pictures taken at better shooting angles can be seen by surgeons to help them perform better in the process of surgery. The laparoscope utilizes the cables to supply power to the ultrasonic motors and the camera, and to transmit the images captured by the camera to the external display device. Once the laparoscope is inserted into the abdominal cavity, only the cables occupy a little space at the abdominal cavity opening, leaving enough room to introduce other surgical instruments for performing the surgery. In this paper, the purpose of manufacturing the laparoscope is to verify the practicability of the designed ultrasonic motors in the laparoscope, so the sealing and disinfection of the laparoscope have not been considered for the time being.

The designed laparoscope has a total length of 110 mm and a maximum diameter of 14.5 mm, which can be inserted into the human body through a trocar with a diameter 15 mm. Parts of the laparoscope are manufactured using a 3D-printing method. The printing material is photosensitive resin, and the printing accuracy is 0.1 mm. The designed laparoscope consists of three modules: (1) magnetically driven module; (2) ultrasonic motor-driving module; (3) camera and lighting module. This section will describe these three modules in detail.

### 3.1. Magnetically Driven Module

**Figure 11:**
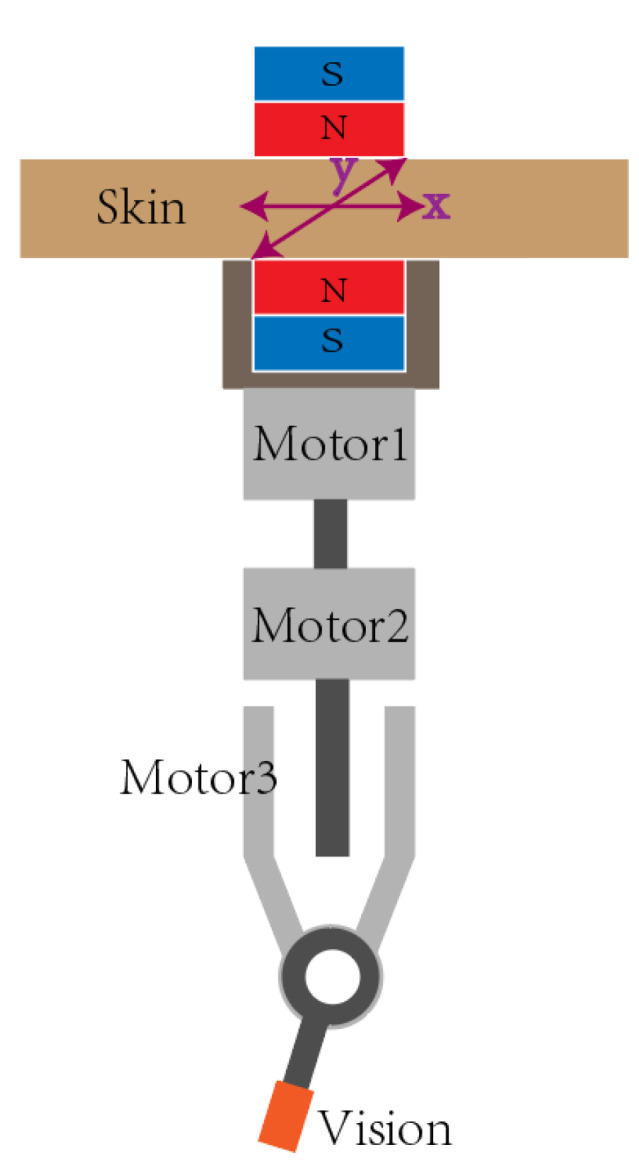
Scheme of the magnetically driven module.

The IPM is located at the top of the laparoscope. Through the magnetic coupling between the EPM and the IPM, the top of the laparoscope is attached to the inner wall of the abdominal cavity. When the laparoscope needs to be moved, the EPM will firstly be moved along the surface of the outer wall of the abdominal cavity, then the laparoscope moves with the EPM. The EPM can drive the laparoscope to move along the X-axis and Y-axis directions, as shown in Figure 11, allowing the laparoscope to reach any position within the abdominal cavity.

### 3.2. Ultrasonic Motor Driving Module

**Figure 12:**
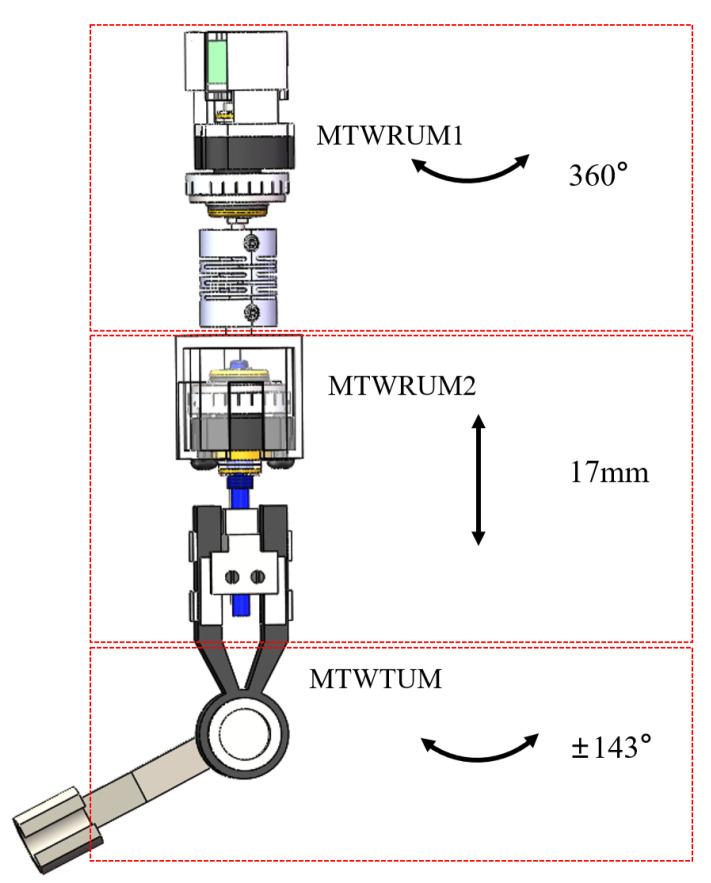
Scheme of the ultrasonic motor driving module.

The ultrasonic motor driving module uses three specially designed ultrasonic motors, including two MTWRUMs and one MTWTUM. The mounting positions of the three ultrasonic motors are illustrated in Figure 12. The MTWRUM1 is located at the top of the laparoscope and can drive the laparoscope to rotate 360${}^{\circ }$. The telescopic joint is set to realize the image zoom. When the output shaft of the MTWRUM2 rotates, the lower part of the laparoscope will be driven to move up or down. The maximum translational distance is 17 mm.

The MTWTUM is located at the lower part of the laparoscope. One end of the camera lens holder is fixedly connected with the MTWTUM output shaft, and rotates synchronously with the MTWTUM output shaft. Due to the limitation of the actual shape of the laparoscope, the maximum tilt angle is 143${}^{\circ }$.

Three ultrasonic motors are used in the laparoscope, and each ultrasonic motor drives one joint of the laparoscope. Therefore, there are three degrees of freedom actuated by the ultrasonic motors, as illustrated in Figure 12. The MTWRUM1 drives the rotary motion, the MTWRUM2 drives the translational motion, and the MTWTUM drives the tilt motion. In addition, the laparoscope can move in both the X and Y directions actuated by the EPM. Therefore, the designed laparoscope has a total of five degrees of freedom.

### 3.3. Camera and Lighting Module

The camera and lighting device are mounted on the camera-lens holder. The camera lens characterizes a optical size of 1/5 inch, a pixel size of 1.75 μm×1.75 μm, a capture rate of 60 fps, a diagonal field of view 60${}^{\circ }$, 2 million resolutions, and the focal length range from 50 mm to infinity. In order to achieve uniform and sufficient illumination, six LED lamps are uniformly arranged around the camera to provide illumination.

## 4. Control of the Magnetically Anchored Laparoscope

### 4.1. Motion Control of the Laparoscope

**Figure 13:**
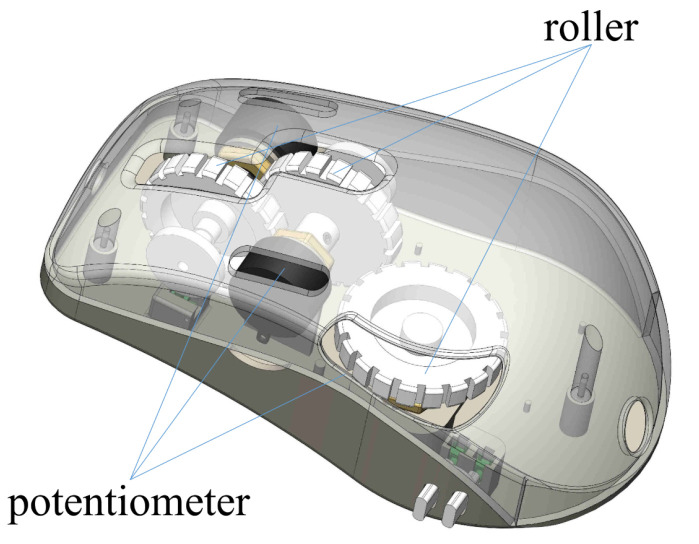
Structural scheme of the mouse-like controller.

To control the motion of the laparoscopic joints, we design a mouse-like controller, whose structure is shown in Figure 13. The mouse-like controller is equipped with three rollers, each of which is fixed with a potentiometer (FCP22E). When surgeons roll the rollers forward or backward, the potentiometers rotates synchronously with the rollers. The potentiometers transmit the sampling values before the rollers rotates (value_original) and after the rollers rotates (value_final) to the microcomputer. When value_final > value_original, the microcomputer controls the ultrasonic motors to rotate forward; when value_final < value_final, the microcomputer controls the ultrasonic motors to rotate backward. The rotational range of the potentiometer is from 0 to 320${}^{\circ }$. When the three potentiometers in the mouse-like controller rotate 320${}^{\circ }$ to the full scale, the first joint of the laparoscope will rotate one circle, the second joint translating 17 mm, and the third joint rotating from −143${}^{\circ }$ to 143${}^{\circ }$, respectively. When the surgeons roate the roller in the laparoscope to drive the potentiometer to rotate, the rotational angle data will be transmitted to the microcomputer (STM32). The microcomputer will calculate the times needed to drive the ultrasonic motors in the laparoscope, which subsequently sends the time data to the motor controllers to drive the ultrasonic motors.

Denoting the rotational angle of the potentiometer as α, the running time of the MTWRUM1 is

(4) $$t_{1}=\frac{\alpha }{320\cdot \omega_{1}}$$

where $\omega_{1}$ (unit: r/s) denotes the rotational speed of the MTWRUM1. As for the driving of translation degree of freedom of the laparoscope, the output shaft of the MTWRUM2 is fixedly connected with a screw-nut pair with a pitch of 0.5 mm, which can convert the rotational motion of the MTWRUM2 into the translational motion of the laparoscope. When the potentiometer rotates an angle ω, the running time of the MTWRUM2 is

(5) $$t_{2}=\frac{\alpha }{320}\cdot \frac{17}{\omega_{2}\cdot 0.5}$$

where $\omega_{2}$ (unit: r/s) denotes the rotational speed of the MTWRUM2. As for the degree of tilt, when the potentiometer rotates an angle ω, the running time of the MTWRUM2 is

(6) $$t_{3}=\frac{\alpha }{320}\cdot \frac{284}{360\cdot \omega_{3}}$$

where $\omega_{3}$ (unit: r/s) denotes the rotational speed of the MTWTUM.

### 4.2. The Suppression of Control Signal Noise

**Figure 14:**
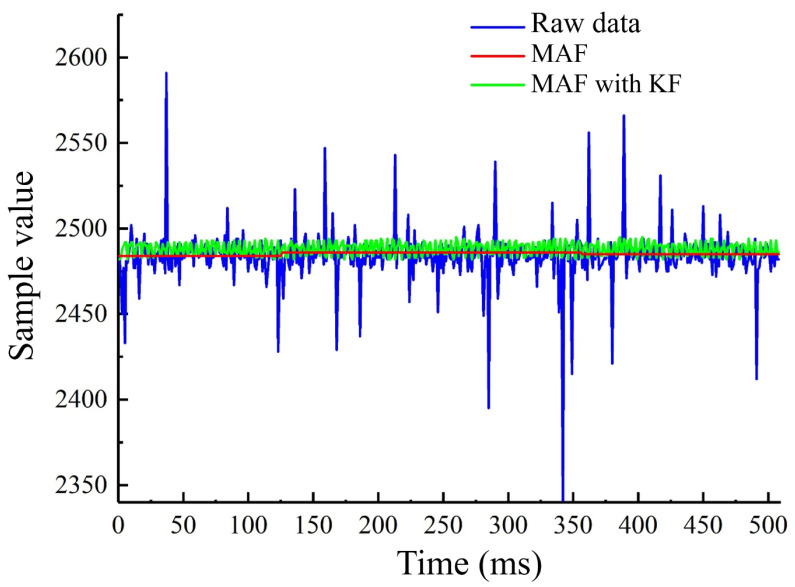
The filtering of the control signal.

When using the laparoscope, hardware factors and hand tremors will introduce errors to the sampling value transmitted to the microcomputer. Median and average filtering and Kalman filtering both are widely used methods for filtering noise. Under the condition of not rotating the roller in the mouse-like controller, we compared the filtering effects of median and average filtering and Kalman filtering combined with median and average filtering to the sampling value. The results are shown in Figure 14. The median and average filtering effectively eliminates the noises with large deviations, but the fluctuation amplitudes of the sampling values are still obvious. After Kalman filtering combined with median and average filtering, the fluctuations of sampling values are further eliminated, and the signals are more stable.

Slight hand tremors occur when the physician is driving the laparoscope, and the errors caused by such tremors should be avoided. The variation of the sampling value received by the microcomputer due to the hand tremors is measured to be within five by the experiment. So we set the microcomputer not to respond when the variation of the sampling value is within five. When the change in the sampling value is greater than five, the microcomputer will send the actuation signal to the motor controller according to the sampling value to drive the motion of the laparoscope.

## 5. Experiments and Results

### 5.1. Torque Output Experiments of the Ultrasonic Motors

**Figure 15:**
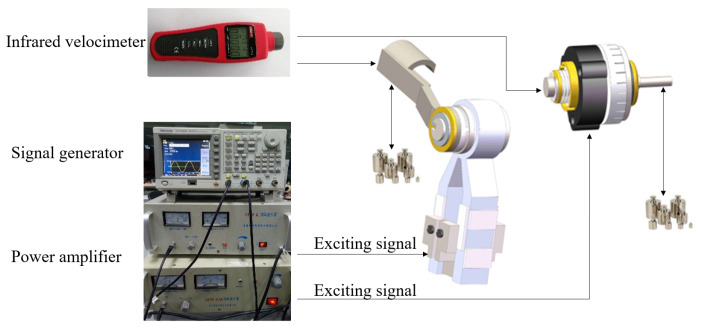
Experimental platform for output characteristics of the ultrasonic motors.

The output torque of the ultrasonic motor is an important index of its working ability, which determines whether the ultrasonic motor can meet the torque requirements for actuating the laparoscope. In this section, output performances of the MTWRUM and the MTWTUM are tested. The experimental platform for testing the output torques of the ultrasonic motors is shown in Figure 15. The signal generator (AFG3022C) sends out two sinusoidal excitation signals with a phase difference of π/2, which are amplified by two power amplifiers (HFVP-83A, HFVA-6), and then serve as excitation signals for the ultrasonic motors. The rotational-speed-measuring instrument is infrared velocimeter (UT372), and the accuracy of this infrared velocimeter is ±0.04% r/min. The speed of the ultrasonic motor will change with the frequency of the excitation signals. In the experiments, the speeds and torques of the ultrasonic motors were tested under the conditions of different-frequency excitation signals. When testing the torque, weights would be suspended at the output ends of the ultrasonic motors to calculate the blocked torque. Each experiment was repeated at least thrice.

**Figure 16:**
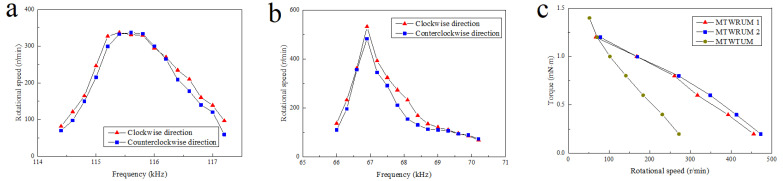
The output characteristics of the ultrasonic motors. (**a**) Rotational speed of the MTWRUM versus excitation voltage frequency; (**b**) rotational speed of the MTWTUM versus excitation voltage frequency; (**c**) output torques versus rotational speed of the ultrasonic motors.

When the voltage of excitation signals is identified as 240 V${}_{\mathrm{pp}}$, the relationship between the excitation signal frequency and the rotational speed of the MTWTUM is shown in Figure 16a. The results show that the working frequency band of the MTWTUM is 114.5∼117.5 kHz. Within the working frequency band, the rotational speed first increases, and then decreases with the increase in the excitation signal frequency. The lowest rotational speed is 87 r/min when the ultrasonic motor rotates clockwise, and 78 r/min when the ultrasonic motor rotates counter-clockwise.

When the voltage of the excitation signal is set at 200 V${}_{\mathrm{pp}}$, the relationship between the excitation signal frequency and the rotational speed of the MTWRUM is shown in Figure 16b. The results demonstrate that the working frequency band of this ultrasonic motor is 66∼70 kHz. Within the working frequency band, the rotational speed of the ultrasonic motor first increases and then decreases with the increase of the excitation signal frequency. The rotational speed changes more sharply compared with the MTWTUM. Under the no-load condition, clockwise rotational speed changes in the range of 73∼482 r/min, and the counter-clockwise rotational speed changes in the range of 68∼532 r/min.

The speeds of the ultrasonic motors change with the frequency of the excitation signals, and thus can be controlled by changing the frequency of the excitation signals. The torques of the ultrasonic motors have an approximately linear relationship with the rotational speeds, as shown in Figure 16c; they will increase with the decrease in the rotational speed. The maximum output torque of the MTWTUM is 1.4 mN·m, and that of the MTWRUM is 1.2 mN·m.

### 5.2. The Movement Experiments of the Laparoscope

**Figure 17:**
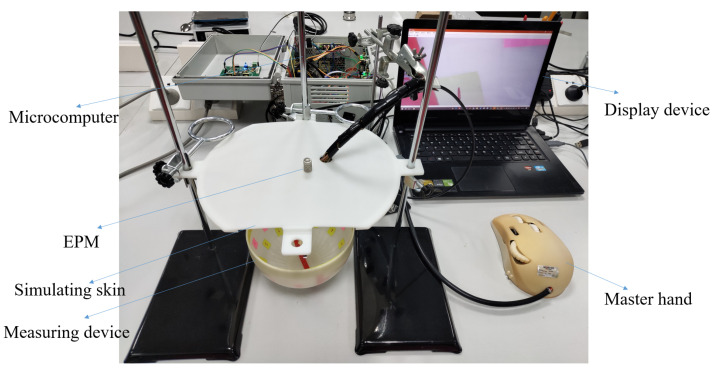
Experimental platform for evaluating the moveability of the laparoscope.

In this section, the movability of the laparoscope was tested. The experimental bench is illustrated in Figure 17. A mouse-like controller was manufactured for controlling the motions of the laparoscopic joints. The bench utilizes a 3D-printed plate to simulate the skin of the human body. The thickness of the plate is 20 mm, which is the skin thickness of a person of medium build [46,47]. An EPM is placed on the plate, and the laparoscope is located under the plate. The laparoscope is attached to the plate surface due to the attraction between the IPM in the laparoscope and the EPM located above the plate. In order to observe the motion of the laparoscope more clearly and accurately, a bowl-shaped measuring device is designed, as shown in Figure 17. The yellow marks are used to record the motion of the laparoscopic rotating joint, and the red marks are used to record the motion of the laparoscopic tilt joint.

**Figure 18:**
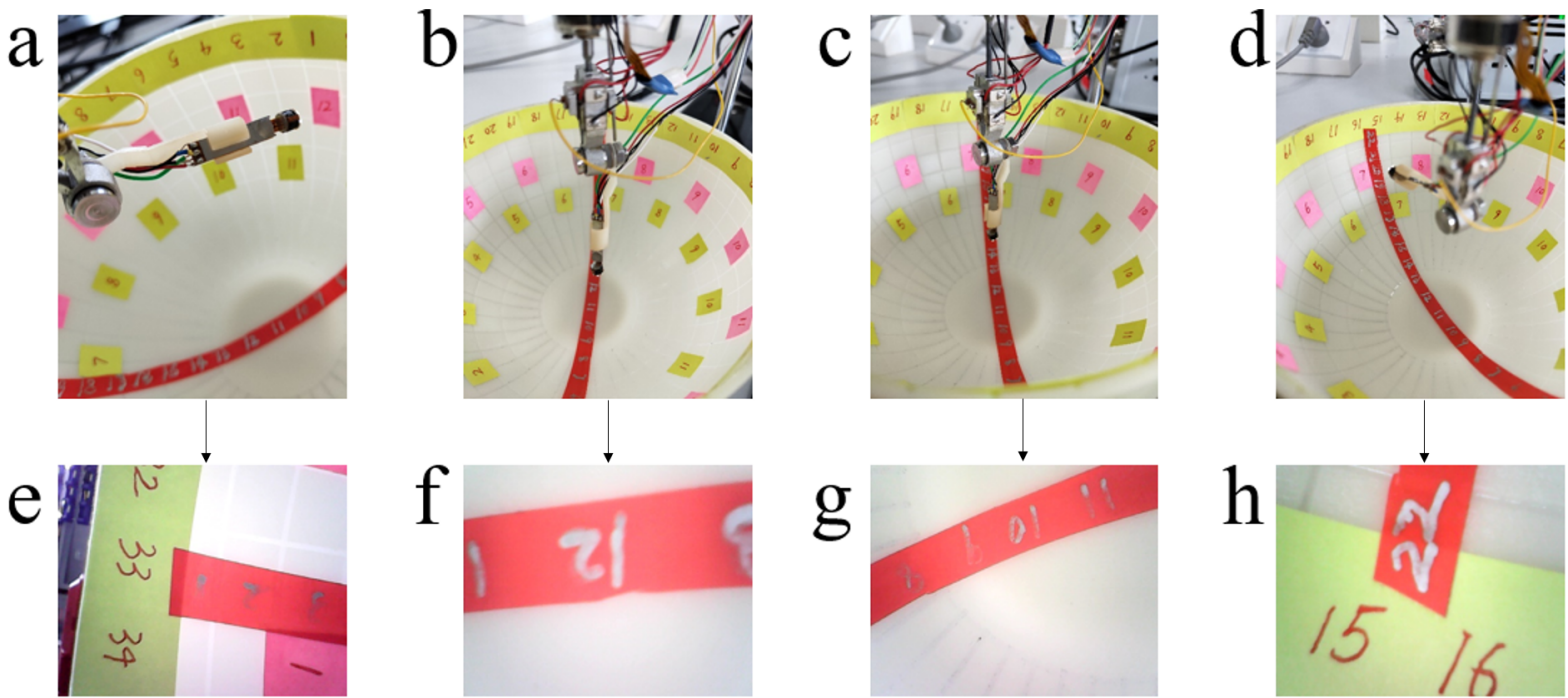
Laparoscopic motions and images. (**a**) The laparoscope is driven to tilt 90${}^{\circ }$; (**b**) the laparoscope is driven to tilt 90${}^{\circ }$ in reverse; (**c**) the laparoscope is driven to extend 17mm; (**d**) the laparoscope is driven to rotate one turn; (**e**) the picture taken when the laparoscope is driven to tilt 90${}^{\circ }$; (**f**) the picture taken when the laparoscope is driven to tilt 90${}^{\circ }$ in reverse; (**g**) the picture taken when the laparoscope is driven to extend 17mm; (**h**) the picture taken when the laparoscope is driven to rotate one turn.

When the laparoscope is not driven to move, it points vertically downward. The laparoscopic tilt joint was driven to tilt 90${}^{\circ }$, and then was driven to reversely tilt 90${}^{\circ }$ to the initial position, as shown in Figure 18a,b. The captured images are shown in Figure 18e,f, respectively. Subsequently, the telescopic joint was driven to stretch 17 mm, as shown in Figure 18c, and the comparison between Figure 18f,g shows that the definition of the captured images changes after the telescopic joint was stretched. Then the tilt joint was driven by 90${}^{\circ }$ until the camera was aligned with the yellow marks at the edge of the measuring device. Subsequently, the rotating joint of the laparoscope was driven to rotate one turn, as shown in Figure 18d, and the captured image is shown in Figure 18h.

Experiments show that each joint of the laparoscope can accurately execute the control commands issued by the mouse-like controller, which shows that the output torques of the three ultrasonic motors are enough to drive the motions of the laparoscope joints.

## 6. Conclusions

To the best of our knowledge, there has been no attempt to use an ultrasonic motor to actuate the motion of the laparoscope. In this paper, the concept of applying ultrasonic motors to the magnetically anchored laparoscope is proposed, and two new ultrasonic motors for mounting on the laparoscope are designed, namely a miniature traveling wave-rotating ultrasonic motor and a miniature traveling wave-tilt ultrasonic motor. Compared with the electromagnetic motor, the ultrasonic motor has the advantages of simple structure, stopping immediately after power failure, being free from magnetic field interference, low speed, and high torque density, etc., which make it more suitable for application in the laparoscope. Using these two kinds of ultrasonic motors, a prototype of the magnetically anchored laparoscope was manufactured. The laparoscope is fixed on the inner wall of the abdominal cavity through the magnetic force between the IPM and the EPM, and the position of the laparoscope can be changed by moving the EPM. Changes in laparoscopic posture are driven by the designed ultrasonic motors, which can realize the movement of the laparoscope in three ways: rotation, translation, and tilt. Output torques of the ultrasonic motors were measured experimentally, and the movability of the laparoscope has been verified, thus proving the practicability of the designed ultrasonic motors in the laparoscope.

In this paper, we focused on the design of the two kinds of ultrasonic motors and the magnetically anchored laparoscope mounted with these two ultrasonic motors. In order to avoid the length of this paper becoming too long, in this paper, we did not analyze the magnetic control to the laparoscope and free from magnetic field interference features of the ultrasonic motors, and so on. In the future, we will analyze these issues in detail, promoting the application of ultrasonic motors in magnetically anchored surgical instruments.

We will consider further reducing the size of the ultrasonic motor under the condition of meeting the torque requirements for driving the laparoscope. A more compact magnetically anchored laparoscope suitable for operating in the human abdominal cavity environment will be designed, and the control system will be optimized, making it easier to operate.

## Figures and Tables

**Table 1:** Mechanical properties of the materials.

Materials	Young’s Modulus (Gpa)	Poisson’s Ratio	Density ($\mathrm{kg}/m^{3}$)
Aluminum alloy	73	0.33	2900
PZT-8	/	/	7600

**Table 2:** Structural dimension parameters of the stator.

Parameters	P1	P2	P3	P4	P5	P6
Numerical value (mm)	4.5	6.9	12	0.3	2	1.5

**Table 3:** Structural dimension parameters of the stator.

Parameters	P1 (mm)	P2 (mm)	P3 (mm)	P4 (mm)	P5 (${}^{\circ }$)	P6 (mm)	P7 (mm)	P8 (mm)
Numerical value	7.5	10.5	5	2.8	36	15.5	55.4	3.5
